# Supplementary material for: An active tethering mechanism controls the fate of vesicles
Source: Nat Commun. 2021 Sep 14;12:5434. doi: 10.1038/s41467-021-25465-y (PMC8440521; doi:10.1038/s41467-021-25465-y)
Supplement: Supplementary file 1 — Supplementary Information [file 41467_2021_25465_MOESM1_ESM.pdf]

Supplementary information

An Active Tethering Mechanism Controls the Fate of Vesicles

Seong J. An, Felix Rivera-Molina, Alexander Anneken[1], Zhiqun Xi[1], Brian McNellis, Vladimir I. Polejaev & Derek Toomre

correspondence to: derek.toomre@yale.edu

This supplementary document includes:

Supplementary Figs. 1 to 12
Supplementary Table 1 and 2

Other supplementary information for this manuscript includes:

Supplementary Movies 1 to 4

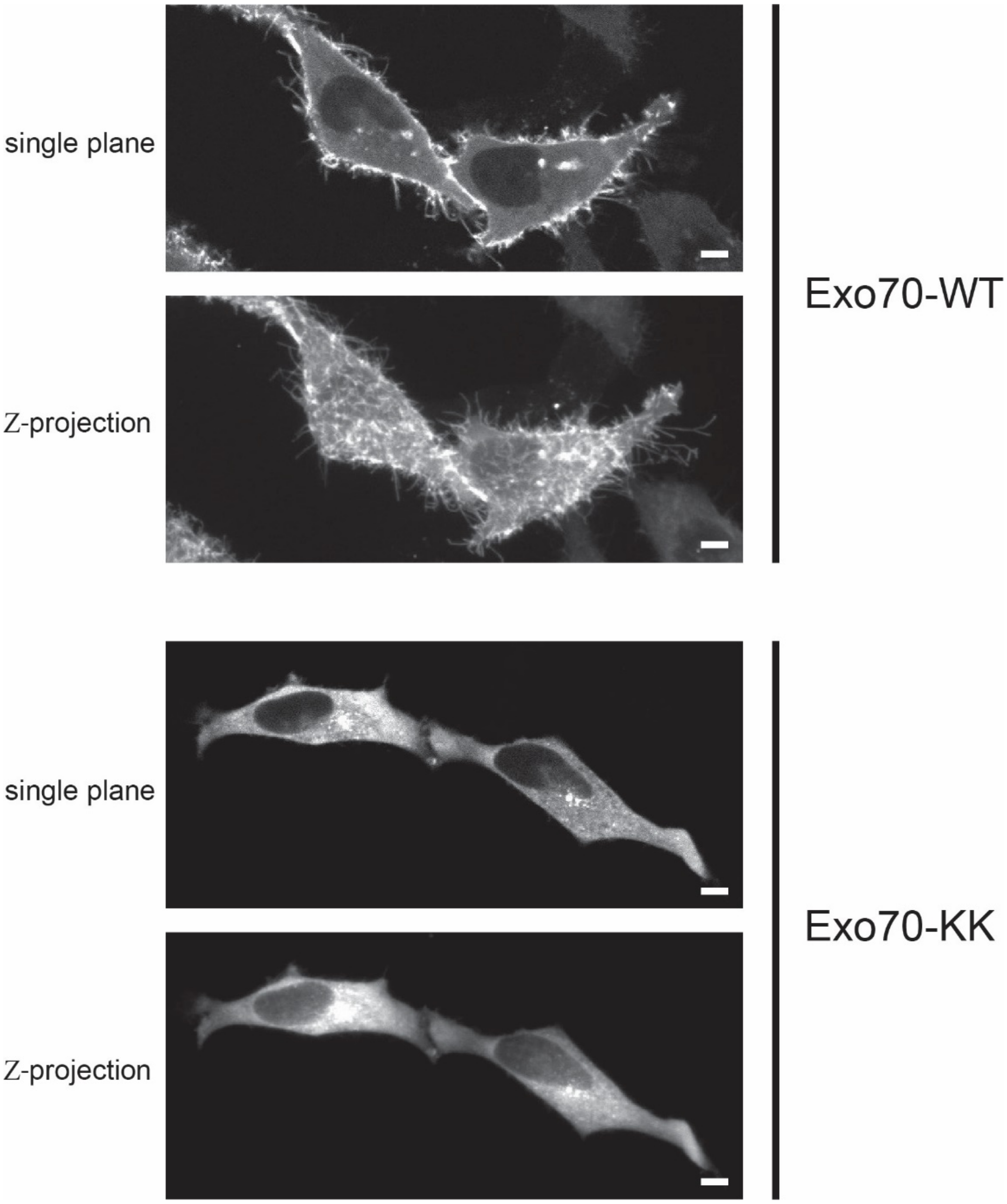

Supplementary Fig. 1. Plasma membrane binding by Exo70-WT but not Exo70-KK. GFP-tagged rat Exo70-WT and -KK were overexpressed without molecular replacement (i.e. without Exo70 KD) in HeLa cells and imaged by confocal microscopy. A single image plane and the Z-projection of the confocal stack is shown for each Exo70 construct. Scale bar, 6 μm.

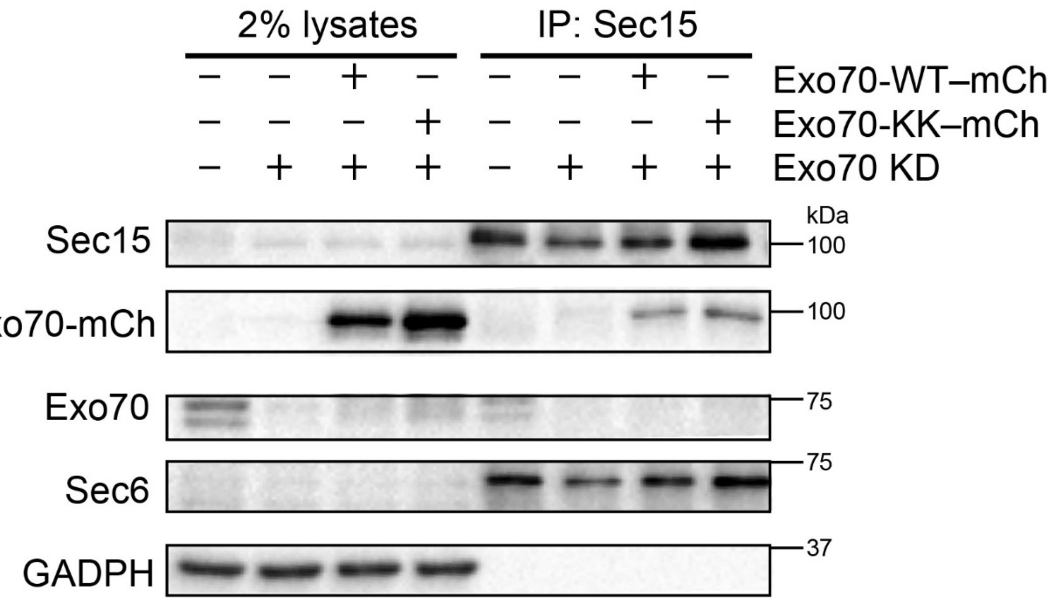

Supplementary Fig. 2. Biochemical reconstitution of exocyst complexes with exogenous Exo70 constructs. Exocyst subunits were co-immunoprecipitated with Sec15 antibody from control or Exo70 KD HeLa cells with or without coexpression of Exo70-mCh constructs.

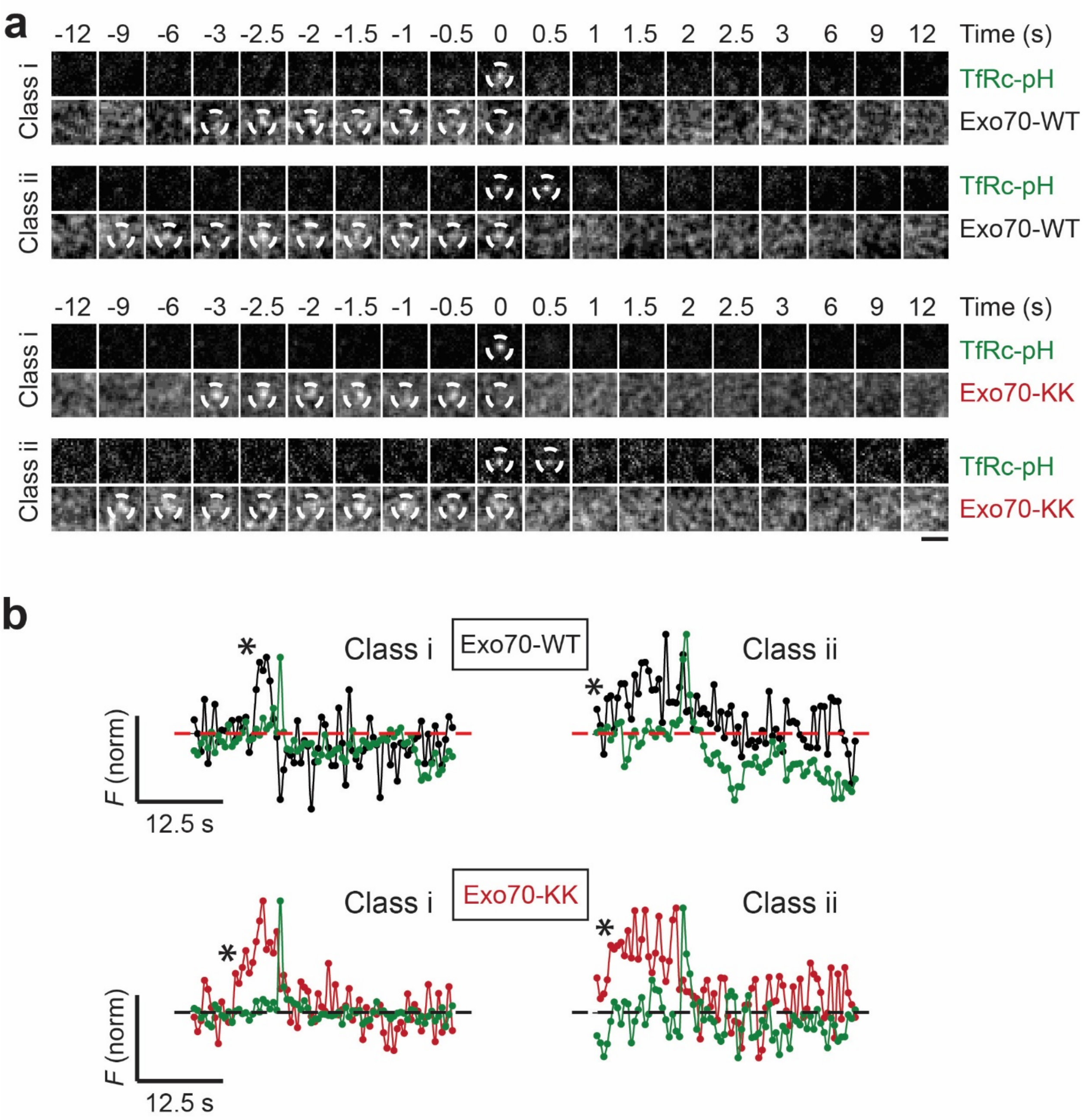

Supplementary Fig. 3. Representative individual short and long tethering events associated with Exo70 WT- and -KK. (**a**) Image sequence of single fusing vesicles labeled with Exo70-WT or -KK. For each Exo70 construct, representative short (labeled "Class i") and long (labeled "Class ii") tethering events are shown. For clarity, an average of 50 frames before fusion was subtracted from each frame of the TfRc-pH channel. Dashed white circles highlight tethering durations in the Exo70 channel and fusion in the TfRc-pH channel. Scale bar, 2 μm. (**b**) Traces of the events in (a) prior to subtraction of the average pre-fusion image for TfRc-pH. Asterisks indicates the start of vesicle tethering. Red and black dashed lines, zero baseline.

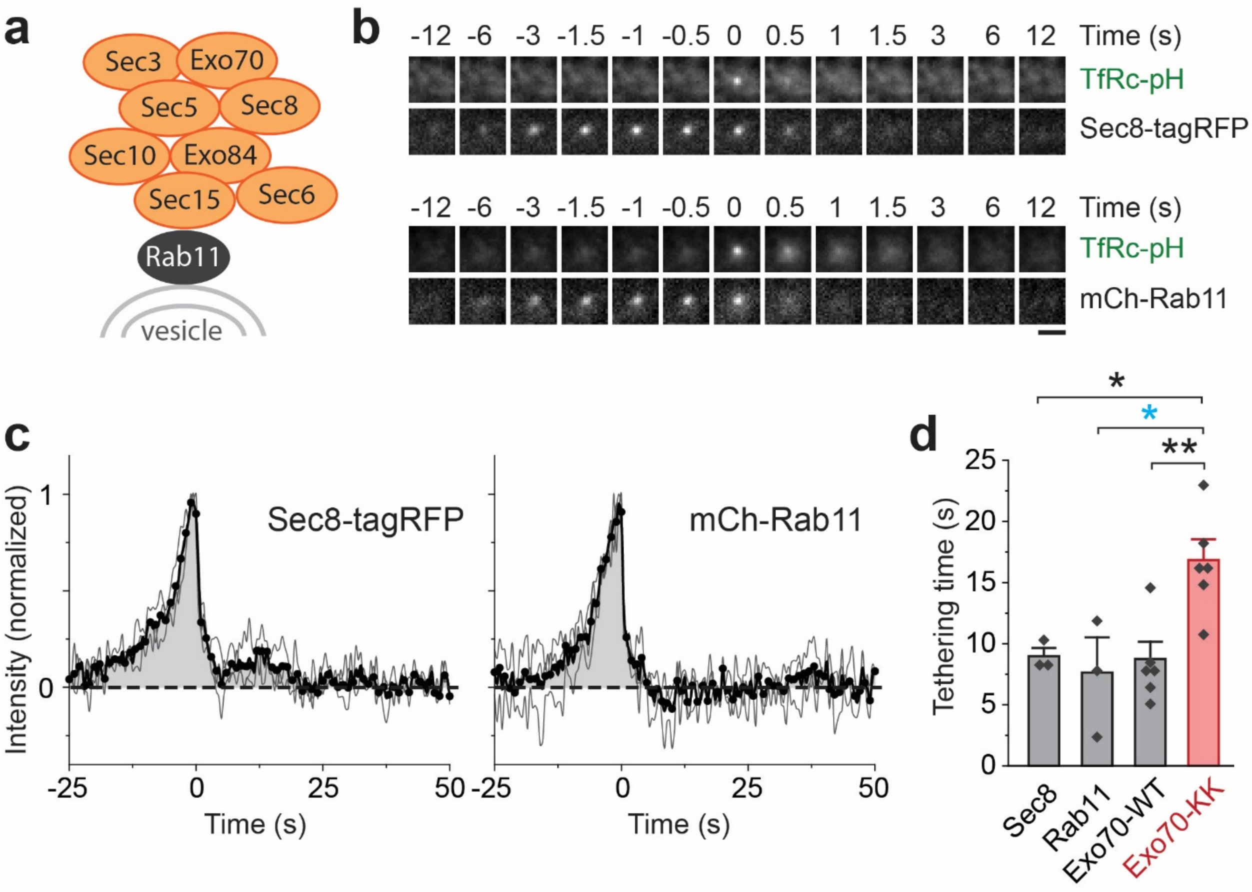

Supplementary Fig. 4. Tethering durations with Sec8 and Rab11 are similar to that of Exo70-WT and but not -KK. (**a**) Schematic representation of the exocyst complex bound to Rab11. The arrangement of exocyst subunits depicted here does not reflect the known connectivity of exocyst subunits[46,47]. (**b**) and (**c**) Sec8 and Rab11 dynamics during tethering and fusion. Average image sequences of fusing vesicles from one cell each (b). Scale bar, 2 µm. Average Sec8 and Rab11 traces, time aligned to fusion (c). Averages (bold line) of cell averages (light lines) are shown. (**d**) The tethering time with Exo70-KK is anomalously long. Tethering times with Exo70-WT and -KK are redisplayed from Fig. 1h (open bars). *n* = 3 cells for Sec8 and Rab11 and *n* = 6 cells for Exo70-WT and -KK. Data represent mean ± SEM (**P* = 0.017, cyan **P* = 0.021, ***P* = 0.0043, two-tailed Student's *t*-test).

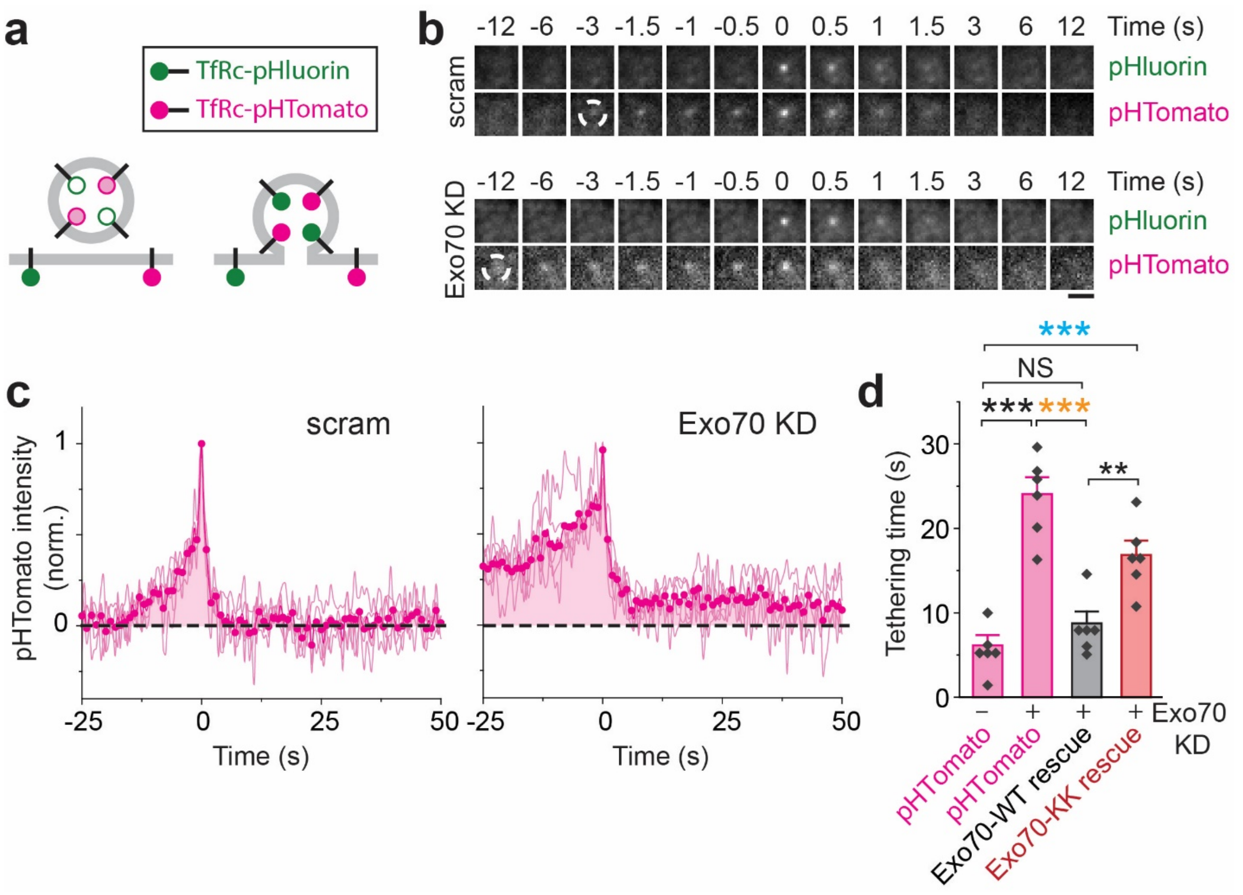

Supplementary Fig. 5. Exo70-WT but not -KK restores the tethering duration after Exo70 KD. (**a**) Schematic of TfRc-pHluorin and -pHTomato fluorescence changes during fusion. The fluorescence of pHTomato, a red pH-sensitive fluorescence protein, is partially quenched (light pink circles) within the acidic environment of endocytic recycling vesicles, which allows vesicle arrival and tethering to be monitored prior to fusion. (**b**) Average image sequence of fusing vesicles from one cell, with or without Exo70 KD (top). Dashed white circle highlights initial tethering. Scale bar, 2 µm. (**c**) TfRc-pHTomato traces (pink), time aligned to fusion. Averages (bold line) of cell averages (light lines) are shown. (**d**) Restoration of short tethering by Exo70-WT but not -KK after Exo70 KD. Tethering times with Exo70-WT and -KK are redisplayed from Fig. 1h (open bars). $n = 6$ cells for each condition. Mean ± SEM (**$P = 0.0043$, ***$P = 1.7\times10^{-5}$, cyan ***$P = 4.5\times10^{-4}$, orange ***$P = 9.3\times10^{-5}$, two-tailed Student's t-test).

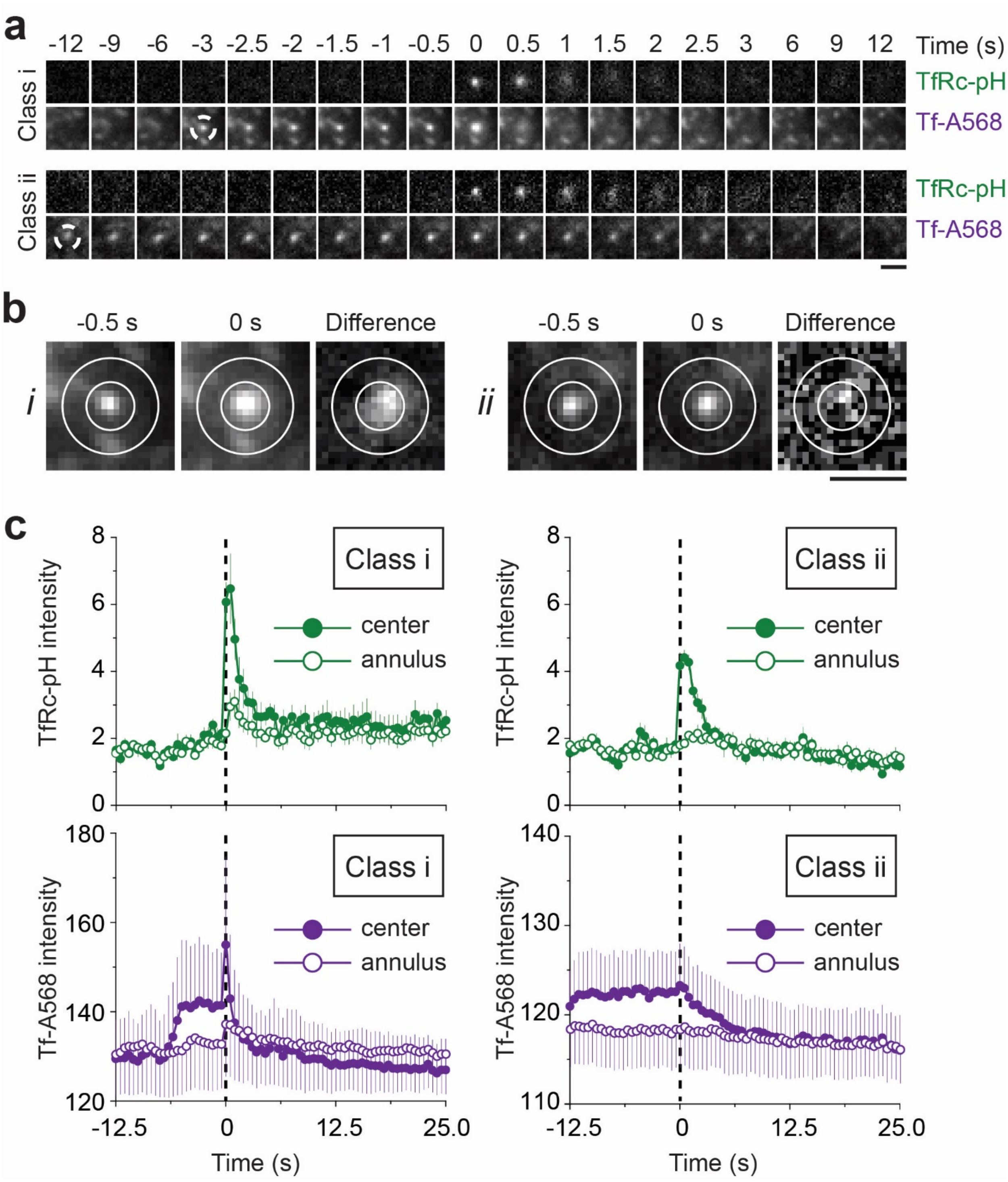

Supplementary Fig. 6. Two classes of fusion events identified by dual imaging of TfRc-pH and Tf-A568. (**a**) Average image sequence of fusing vesicles labeled with TfRc-pH and Tf-

A568. Vesicles were grouped into two classes, based on the spreading of Tf-A568 fluorescence during fusion: spreaders (labeled "Class *i*") and non-spreaders (labeled "Class *ii*"). A subset of vesicles (*n* = 7) from Fig. 1f (25 mM HEPES condition) was averaged for each class. For clarity, an average of 50 frames before fusion was subtracted from each frame of the TfRc-pH channel. Dashed white circles highlight initial tethering. Note the faster diminishment of TfRc-pH compared to that of Tf-A568 in the Class ii average. Scale bar, 2 μm. (**b**) Tf-A568 fluorescence at the moment of fusion. Difference images were obtained by subtracting the last frame before fusion (–0.5 s) from the first frame of fusion (0 s). Concentric circles are used to measure the fluorescence enclosing a vesicle (center) and its surrounding area (annulus). Note that a fluorescence increase within the annulus is seen only in the Class i event. Difference images are displayed at a different contrast setting from their parent images to better show fluorescence spreading or nonspreading. Scale bar, 2 μm. (**c**) Plots of fluorescence intensities within the central and annular regions of the averaged events in (a), prior to subtraction of the average pre-fusion image for TfRc-pH. Mean ± SEM.

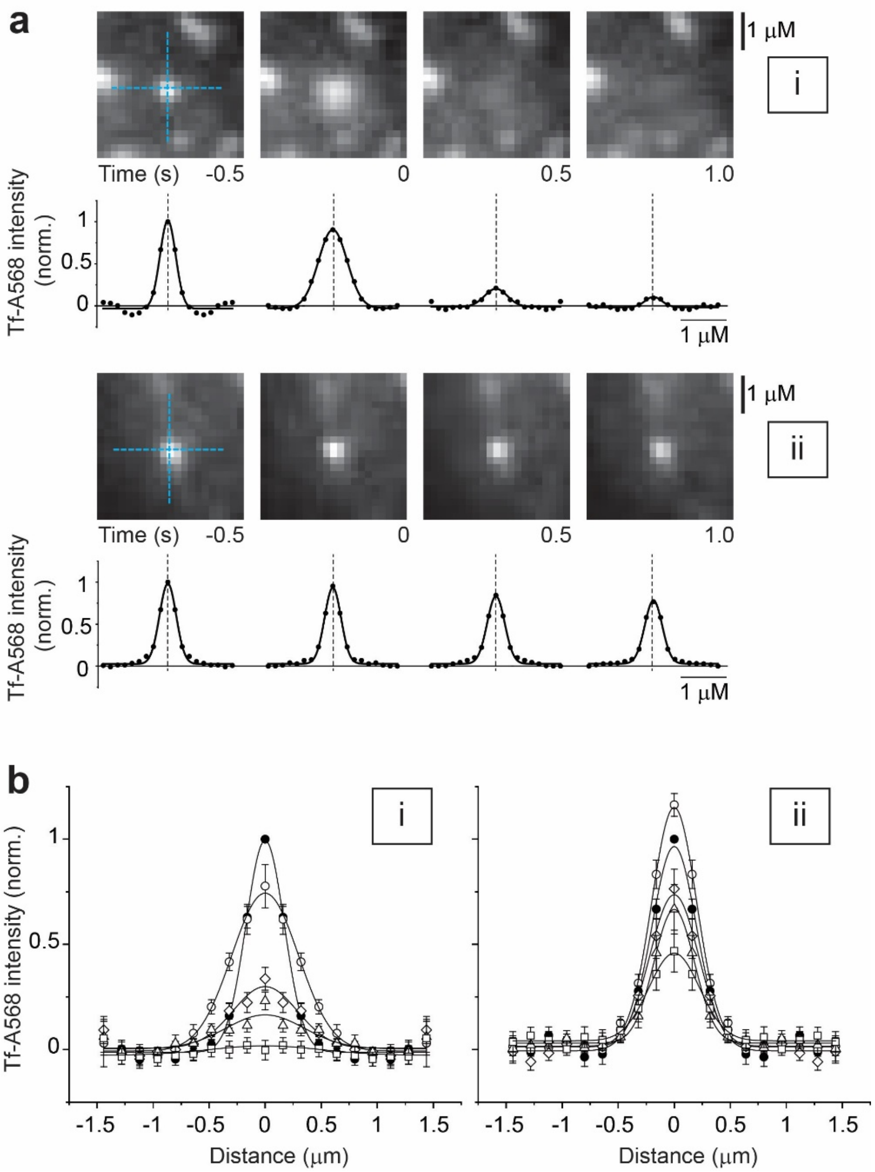

Supplementary Fig. 7. Diffusion of vesicle cargo for Class i but not Class ii fusion events. (**a**) Consecutive images of Tf-A568 immediately before and during exocytosis for a single Class i (short tethering/fast fluorescence decay) and Class ii (long tethering/slow fluorescence decay) event (top). For each timepoint, line-intensity profiles (cyan dashed lines) were averaged and fitted with Gaussian functions (bottom). (**b**) Average fluorescence profiles at selected timepoints relative to fusion (filled circles, −0.5 s; open circles, 0 s; diamonds, 0.5 s; triangles, 1.0 s; squares, 1.5 s). n = 12 vesicles for both Class i and Class ii fusion events. Mean ± SEM. Note the faster decrease in the amplitude of the fluorescence profiles with Class i events.

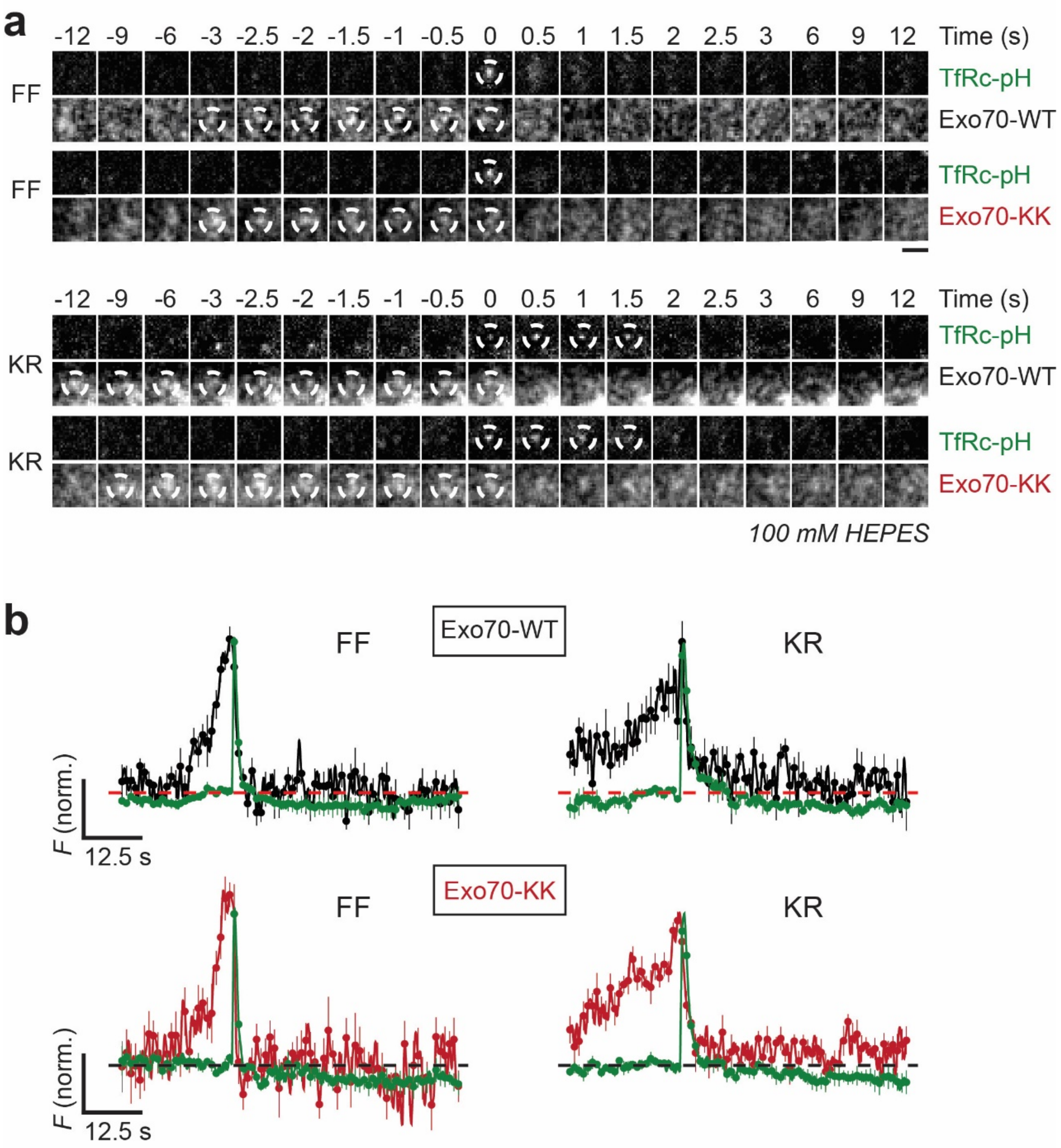

Supplementary Fig. 8. Representative individual FF and KR events associated with Exo70 WT- and -KK. (**a**) Image sequence of single fusing vesicles labeled with Exo70-WT or -KK in 100 mM HEPES. For each Exo70 construct, representative FF and KR events are shown. For clarity, an average of 50 frames before fusion was subtracted from each frame of the TfRc-pH channel. Dashed white circles highlight tethering durations in the Exo70 channel and fusion in the TfRc-pH channel. Scale bar, 2 µm. (**b**) Average Exo70 and TfRc-pH traces during FF and KR, prior to subtraction of the average pre-fusion image for TfRc-pH. *n* = 6 cells for Exo70-WT and *n* = 7 cells for Exo70-KK. Mean ± SEM. Red and black dashed lines, zero baseline.

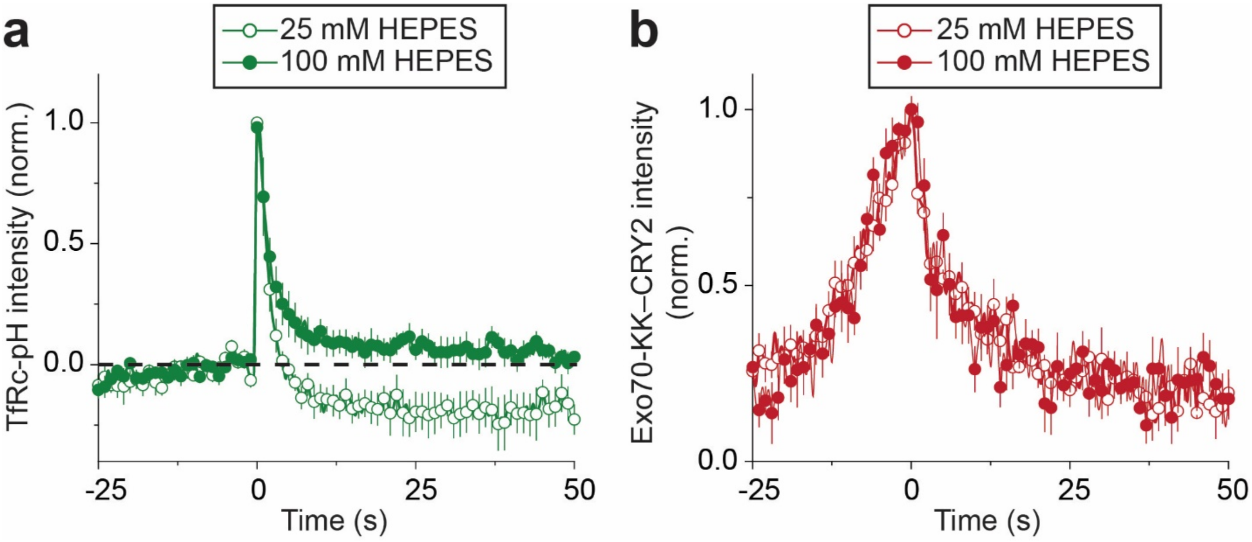

Supplementary Fig. 9. Exo70 optogenetics produces a TfRc-pH signal that remains elevated after fusion in 100 mM HEPES. (**a**) TfRc-pH and (**b**) Exo70-KK–CRY2 traces, time aligned to fusion. Averages (bold line) of cell averages (light lines) are shown. $n = 5$ cells for 25 and 100 mM HEPES. Mean ± SEM. Note that TfRc-pH does not decay below the zero baseline (dashed line) in 100 mM HEPES.

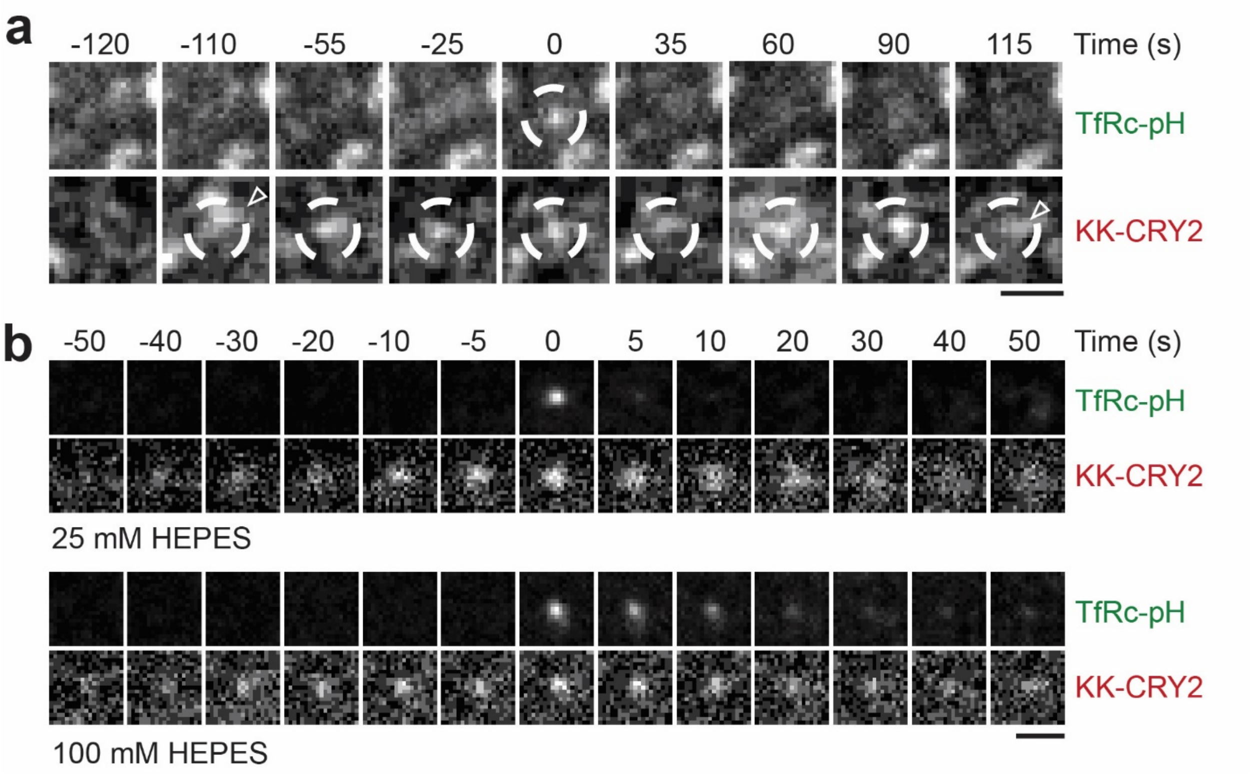

Supplementary Fig. 10. Low-frequency Exo70 optogenetics promotes KS instead of FF. (**a**) Images of a single vesicle undergoing long tethering during low frequency (0.2 Hz) activation. Scale bar, 2 µm. (**b**) Average image sequence of fusing vesicles (from one cell) during low frequency activation with or without high extracellular HEPES concentration. For clarity, an average of 10 frames before fusion was subtracted from each frame of the TfRc-pH channel. Note that lateral movement of vesicles before and after fusion causes blurring of the averaged Exo70-KK–CRY2 spot at timepoints farther away from t = 0 s. Scale bar, 2 µm.

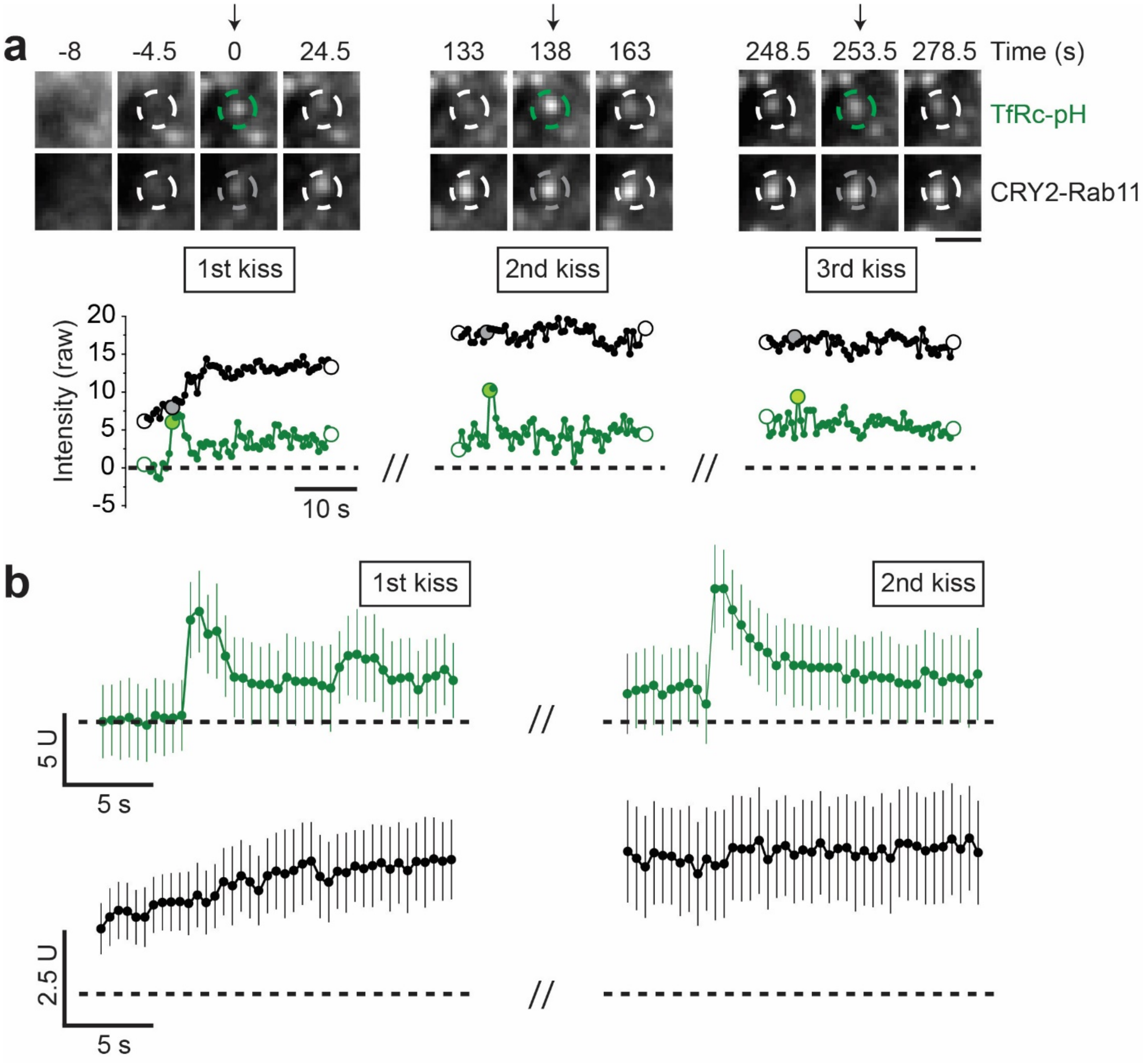

Supplementary Fig. 11. Repeated kissing by vesicles with Rab11 optogenetics. (**a**) Images of a single vesicle repeatedly kissing. Arrows indicate fusion onset. Colored circles in the traces (bottom) correspond to dashed circles in the image frames (top). Scale bar, 2 µm. (**b**) Pooled averages of TfRc-pH and CRY2-Rab11 for vesicles that kiss at least twice (n = 13 events). Mean ± SEM. Dashed lines, zero baseline.

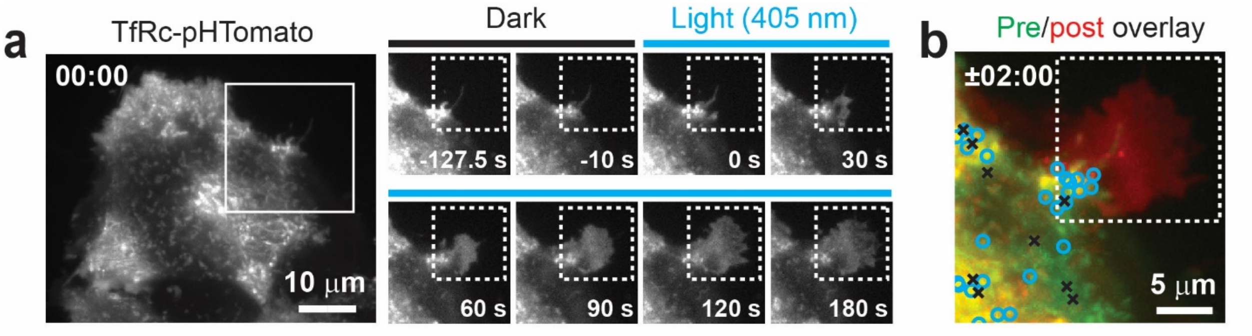

Supplementary Fig. 12. Membrane expansion is triggered during but not before 405-nm light stimulation. TIRFM of TfRc-pHTomato in a typical Exo70 optogenetics cell. (**a**) Image sequence (right) of region outlined by solid box. Dashed box in image sequence highlights expanding membrane. Note that Exo70-KK–CRY2 without mCherry was used in these experiments. (**b**) Map of FF events during the first two min before (crosses) and after (circles) light stimulation.

Figure	Experiment	Method	Condition	# of vesicles	# of cells
**1a**	Endogenous Exo70 localization	Immunofluorescence	scram<br>Sec15 KD	N/A<br>N/A	11<br>12
**1b, c**	Exo70-WT and -KK dynamics during tethering and fusion	TIRFM	Exo70-WT–mCh<br>Exo70-KK–mCh	98<br>117	5<br>5
**S4**	Tethering time comparisons	TIFRM	Sec8-tagRFP<br>mCh-Rab11	68<br>71	3<br>3
**S5**	Tethering time rescue by Exo70-WT	TIRFM	TfRc-pHTomato (scram)<br>TfRc-pHTomato (Exo70 KD)	71<br>53	5<br>5
**1f**	Demonstration of FF and KR by recycling vesicles	TIRFM	Tf-A568<br>Tf-A568 (100 mM HEPES)	65<br>76	9<br>17
**1g, h**	Fusion modes with Exo70-WT and -KK	TIRFM	Exo70-WT–mCh (100 mM HEPES)<br>Exo70-KK–mCh (100 mM HEPES)	146<br>164	6<br>7
**2b–f**	Rescue of FF by Exo70-KK–Cry2	TIRFM/ Optogenetics	-CIB, 100 mM HEPES, 1.5W/cm^2<br>+CIB, 1.5W/cm2	143<br>224	4<br>5
**3b–f**	Rescue of FF by Exo70-KK–mCh-SspB	TIRFM/ Optogenetics	-CIB, 100 mM HEPES, 1.5W/cm^2<br>+CIB, 100 mM HEPES, 1.5W/cm^2	149<br>74	4<br>4
**4a**	Effect of light dosage on fusion modes with Exo70-KK–Cry2	TIRFM/ Optogenetics	+ CIB, 100 mM HEPES, 0.23 W/cm^2<br>+ CIB, 100 mM HEPES, 1.5 W/cm^2<br>+CIB, 100 mM HEPES, 2.6 W/cm^2	137<br>160<br>290	5<br>5<br>5
**4b–d**	Low frequency activation of Exo70-KK–CRY2	TIRFM/ Optogenetics	+CIB, 1.5 W/cm^2, 0.2 Hz<br>+CIB, 100 mM HEPES, 1.5 W/cm^2, 0.2 Hz	52<br>36	2<br>1
**4e, f**	Effect of Exo70 KD on fusion mode	TIRFM	Sec8-tagRFP (scram, 100 mM HEPES)<br>Sec8-tagRFP (Exo70 KD)	159<br>127	5<br>5
**5b–d**	CRY2-Rab11 optogenetics	TIRFM/ Optogenetics	mCh-CRY2–Rab11 (scram, 1.5 W/cm^2)<br>mCh-CRY2–Rab11 (Exo70 KD, 1.5 W/cm^2)	150<br>167	5<br>4
**5e–g**	Fusion modes with CRY2-Rab11	TIRFM/ Optogenetics	Exo70 KD, 100 mM HEPES, 1.5 W/cm^2<br>Exo70 KD, 100 mM HEPES, 2.6 W/cm^2	118<br>98	5<br>4
**6a–d**	Membrane expansion induced by Exo70-KK–CRY2	TIRFM/ Optogenetics	-CIB, 2.6 W/cm^2<br>+CIB, 0.23 W/cm^2<br>+CIB, 2.6 W/cm^2	N/A<br>N/A<br>N/A	11<br>11<br>11
**S12**	405-nm activation of Exo70-KK–CRY2	TIRFM/ Optogenetics	Exo70-KK–CRY2 (+CIB)	N/A	2
**6e**	Actin remodeling induced by Exo70-KK–CRY2	TIRFM/ Optogenetics	LifeAct-GFP (Exo70-KK–CRY2-mCh, 2.6 W/cm^2)	N/A	5

Supplementary Table 1. Number and sample size of experiments in the main and supplementary figures. Unless indicated, optogenetics experiments were performed with 2 Hz, 488-nm activation.

Exo70-mCherry	Forward<br>Reverse	GAATTCTGGCCACCATGATTCCCCCGCAGGAGGCTTC<br>CGGTACCGTAGAACCACCAGCAGAGGTGTCGAAAAGGCGATCG
Exo70-KK mutagenesis	Forward<br>Reverse	CACCAAAAACCCGGAGGCCTACATCGCGTACCGCGTGGAGCAGG<br>CCTGCTCCACGCGGTACGCGATGTAGGCCTCCGGGTTTTTGGTG
Exo70-KK-CRY2-mCherry	Forward<br>Reverse	GCTAGCGCCACCATGATTCCCCCGCAGGAGGCTTC<br>CTCGAGAGAACCACCAGAACCACCAGAACCACCAGAACCACCAGCAGAGGTGTCGAAAAGGCGATCG
mCherry-Rab11a	Forward<br>Reverse	AATTCTGGAGGTTCTATGGGCACCCGCGACGACGAGTAC<br>GGTACCTCAGATGTTCTGACAGCACTGCACCTTTGGCTTG
mCherry-CRY2-Rab11a	Forward<br>Reverse	CGATCGGGTGGTTCTATGGGCACCCGCGACGACGAGTAC<br>GGTACCTCAGATGTTCTGACAGCACTGCACCTTTGGCTTG
TfRc-pHTomato	Forward<br>Reverse	ACCGGTCGCCACCATGGTGAGCAAGGGCGAGGAGAATAAC<br>TCTAGATCACTTGTACAGCTCGTCCATGCCGC
Exo70-KK-mCherry-SspB	Forward<br>Reverse	TCAAGCTTATGATTCCCCCGCAGGAGGCTTC<br>CGACCGGTGGAGAACCACCAGAACCACCAGCAGAGGTGTCGAAAAGGCGATC

Supplementary Table 2. Complete list of primers used
