# Supplementary material for: An active tethering mechanism controls the fate of vesicles
Source: Nat Commun. 2021 Sep 14;12:5434. doi: 10.1038/s41467-021-25465-y (PMC8440521; doi:10.1038/s41467-021-25465-y)
Supplement: Supplementary file 3 — Description of Additional Supplementary Files [file 41467_2021_25465_MOESM3_ESM.pdf]

Description of Additional Supplementary Files

File Name: Supplementary Movie 1
Description: Average tethering and fusion with Exo70-WT and -KK. TfRc-pH (left) and Exo70-mCh constructs (right). Each movie is an average of 23 and 26 fusion events from an Exo70-WT and -KK cell, respectively. Note the longer tethering duration with Exo70-KK. Playback speed, 15 fps.

File Name: Supplementary Movie 2
Description: Exo70-KK–CRY2 dynamics. Exo70-KK-CRY2 activation with (bottom) or without (top) coexpression of CIB. Note that some Exo70-KK-CRY2 vesicles become immobilized in the +CIB, but not – CIB, movie. Playback speed, 30 fps.

File Name: Supplementary Movie 3
Description: Membrane expansion induced by Exo70-KK–CRY2 activation. A cell expressing TfRc-pHTomato and Exo70-KK-CRY2 is activated with 405-nm light starting at t = 0 s, indicated by an arrowhead. Playback speed, 30 fps.

File Name: Supplementary Movie 4
Description: Filopodia elongation induced by Exo70-KK–CRY2 activation. Two regions (“i” and “ii”) from a cell expressing Lifeact-GFP and Exo70-KK-CRY2 are shown during activation with 488-nm light. Playback speed, 30 fps.
