# Supplementary material for: An active tethering mechanism controls the fate of vesicles
Source: Nat Commun. 2021 Sep 14;12:5434. doi: 10.1038/s41467-021-25465-y (PMC8440521; doi:10.1038/s41467-021-25465-y)
Supplement: Supplementary file 8 — Reporting Summary [file 41467_2021_25465_MOESM8_ESM.pdf]

nature research

Corresponding author(s): Derek Toomre

Last updated by author(s): Jun 25, 2021

Reporting Summary

Nature Research wishes to improve the reproducibility of the work that we publish. This form provides structure for consistency and transparency in reporting. For further information on Nature Research policies, see our Editorial Policies and the Editorial Policy Checklist.

Statistics

For all statistical analyses, confirm that the following items are present in the figure legend, table legend, main text, or Methods section.

n/a	Confirmed	
[ ]	[x]	The exact sample size (*n*) for each experimental group/condition, given as a discrete number and unit of measurement
[ ]	[x]	A statement on whether measurements were taken from distinct samples or whether the same sample was measured repeatedly
[ ]	[x]	The statistical test(s) used AND whether they are one- or two-sided<br>*Only common tests should be described solely by name; describe more complex techniques in the Methods section.*
[x]	[ ]	A description of all covariates tested
[x]	[ ]	A description of any assumptions or corrections, such as tests of normality and adjustment for multiple comparisons
[ ]	[x]	A full description of the statistical parameters including central tendency (e.g. means) or other basic estimates (e.g. regression coefficient) AND variation (e.g. standard deviation) or associated estimates of uncertainty (e.g. confidence intervals)
[ ]	[x]	For null hypothesis testing, the test statistic (e.g. F, t, r) with confidence intervals, effect sizes, degrees of freedom and P value noted<br>*Give P values as exact values whenever suitable.*
[x]	[ ]	For Bayesian analysis, information on the choice of priors and Markov chain Monte Carlo settings
[x]	[ ]	For hierarchical and complex designs, identification of the appropriate level for tests and full reporting of outcomes
[ ]	[x]	Estimates of effect sizes (e.g. Cohen's *d*, Pearson's *r*), indicating how they were calculated

Our web collection on statistics for biologists contains articles on many of the points above.

Software and code

Policy information about availability of computer code

Data collection	Fluorescence microscopy images were acquired using iQ imaging software version 1.10.1 (Andor Technology) and SoftWorX software version 6.5.2 (Applied Precision).
Data analysis	Fluorescence microscopy images were analyzed using MetaMorph software version 7.1.2 (Molecular Devices), Excel (Microsoft) and Origin (OriginLab).

For manuscripts utilizing custom algorithms or software that are central to the research but not yet described in published literature, software must be made available to editors and reviewers. We strongly encourage code deposition in a community repository (e.g. GitHub). See the Nature Research guidelines for submitting code & software for further information.

Data

Policy information about availability of data

All manuscripts must include a data availability statement. This statement should provide the following information, where applicable:

- Accession codes, unique identifiers, or web links for publicly available datasets
- A list of figures that have associated raw data
- A description of any restrictions on data availability

The data that support the findings of this study are available from the authors on reasonable request. The accession code for mouse Exo70 used in the manuscript is BC028927 (GenBank accession [https://www.ncbi.nlm.nih.gov/nuccore/BC028927]).

Field-specific reporting

Please select the one below that is the best fit for your research. If you are not sure, read the appropriate sections before making your selection.

- [x] Life sciences
- [ ] Behavioural & social sciences
- [ ] Ecological, evolutionary & environmental sciences

For a reference copy of the document with all sections, see nature.com/documents/nr-reporting-summary-flat.pdf

Life sciences study design

All studies must disclose on these points even when the disclosure is negative.

Sample size	Sample size reflected the number of times an experiment was repeated to deem that an experimental finding was reproducible, which generally reflected the robustness of a particular result.
Data exclusions	Imaging data were excluded only if images were of low quality (i.e. too noisy) or visually marred by some factor (e.g., a contaminating nearby signal in single fusion event experiments) and could not justifiably included in a dataset.
Replication	Reproducibility of experimental findings was deemed positive by multiple (3 or more), independent attempts at replication.
Randomization	No randomization was attempted as it was not necessary for this field of study.
Blinding	Authors were not blinded to the collection and analysis of images because these tasks, due to their sophisticated nature, were required to be performed by the same authors.

Reporting for specific materials, systems and methods

We require information from authors about some types of materials, experimental systems and methods used in many studies. Here, indicate whether each material, system or method listed is relevant to your study. If you are not sure if a list item applies to your research, read the appropriate section before selecting a response.

Materials & experimental systems

n/a	Involved in the study	
[ ]	[x]	Antibodies
[ ]	[x]	Eukaryotic cell lines
[x]	[ ]	Palaeontology and archaeology
[x]	[ ]	Animals and other organisms
[x]	[ ]	Human research participants
[x]	[ ]	Clinical data
[x]	[ ]	Dual use research of concern

Methods

n/a	Involved in the study	
[x]	[ ]	ChIP-seq
[x]	[ ]	Flow cytometry
[x]	[ ]	MRI-based neuroimaging

Antibodies

Antibodies used	The Exo70 (clone 70X13F3; catalog# ED2001; lot# 012916) and Sec15 (clone 15s2G6; catalog# ED2003; lot# 012916) antibodies are from Kerafast. The GAPDH antibody (clone14C10; catalog# 2118S; lot# 10) is from Cell Signaling. The Sec6 antibody is a pooled aliquot from monoclonal Ab generated by the Scheller lab and referenced in the manuscript. Goat anti-mouse labeled with Atto647N is from Sigma (50185-1ML-F).
Validation	-Validation for the commercial antibodies is provided on the following web pages:<br>Anti-Exo70 (ED2001; https://www.kerafast.com/item/579/anti-exocyst-complex-exo70-exoc7-subunit-70x13f3-antibody)<br>Anti-Sec15 (ED2003); https://www.kerafast.com/item/581/anti-exocyst-complex-sec15-exoc6-subunit-15s2g6-antibody)<br>Anti-GAPDH (2118S; https://www.cellsignal.com/products/primary-antibodies/gapdh-14c10-rabbit-mab/2118)<br>Goat-anti-mouse-Atto647N (50185-1ML-F; https://www.sigmaaldrich.com/US/en/product/sigma/50185; this antibody is cited in 85 papers on the manufacturer's website)<br><br>-Validation for anti-Sec6 antibody is described in Hsu, S. et al. (Hsu, S. et al. Subunit composition, protein interactions, and structures of the mammalian brain sec6/8 complex and septin filaments. Neuron 20, 1111–1122, 1998). This antibody recognizes a single band corresponding to its antigen in rat brain homogenate.

Eukaryotic cell lines

Policy information about cell lines

Cell line source(s)	HeLa cells are from ATCC. Stable TfRc-pH and double-stable Sec8-tagRFP/Sec8 KD cell lines were generated with these cells.

Authentication	HeLa cells were obtained directly from ATCC.
Mycoplasma contamination	Cell line was tested negative for mycoplasma.
Commonly misidentified lines (See ICLAC register)	No commonly misidentified lines were used in this study.
